# Child Abuse and Neglect Awareness Among Healthcare Students in Saudi Arabia

**DOI:** 10.7759/cureus.51470

**Published:** 2024-01-01

**Authors:** Hawa M Alabdulaziz, Teif H Dawood, Shahad F Baswaid, Khawlah A Zabarmawi, Haneen A Baswid, Nasreen A Baloush

**Affiliations:** 1 Maternity and Children Department/Pediatric Nursing, Faculty of Nursing, King Abdulaziz University, Jeddah, SAU; 2 Faculty of Nursing, King Abdulaziz University, Jeddah, SAU

**Keywords:** saudi arabia, healthcare students, child neglect, child abuse, awareness

## Abstract

Background

Healthcare workers are responsible for dealing with pediatric victims of violence. While the awareness and reporting of suspected cases are rising, there is a lack of research that proves the awareness, knowledge, and attitude of healthcare students.

Objectives

We evaluated the awareness of child abuse and neglect and examined the demographic factors associated with them.

Participants and setting

The study was conducted at King Abdulaziz University in Jeddah with a sample of 237 undergraduate healthcare students.

Methods

We utilized the quantitative design and surveyed a convenience sample of 237 healthcare students from March to May 2021. The survey contains questions on child abuse and neglect.

Results

The respondents’ overall mean on the scale was 82.90 (SD = 14.00). The students rated the subscale “Actions considered as child neglect” as the highest (M = 4.18, SD = 1.08), followed by “Actions considered as child abuse” (M = 4.04, SD = 1.21), “Reasons for under-reporting child abuse and neglect cases in Saudi Arabia” (M = 3.34, SD = 0.68), and “Knowledge regarding child abuse and neglect” (M = 3.13, SD = 0.53). The analyses showed that gender (t= -3.88, p< 0.001) and year level (F= 5.98, p= 0.003) were associated with the students' "knowledge regarding child abuse and neglect."

Conclusion

The findings revealed a good awareness of child maltreatment among healthcare students. However, the students reported a lower awareness of the different reasons for under-reporting child violence cases in Saudi Arabia.

## Introduction

Child abuse and neglect occur worldwide. The first incidence of child abuse in Saudi Arabia was reported in the medical literature in 1990 [1]. Subsequently, the government committee on protecting children's rights in the country was established in 1994. However, we cannot be sure that this is the first case or that all cases were subsequently reported and treated. A study by Alnasser et al. (2017) identified issues, such as inadequate awareness, inappropriate behaviors, and misconceptions, to consider in future programs and policies regarding child maltreatment in the country [2].

Reporting child maltreatment cases to the responsible authorities is mandatory for healthcare employees to deliver prompt intervention to victims and prevent further damage [3]. However, many studies showed a lack of awareness regarding child abuse and neglect among healthcare providers who often interact with children in hospitals [4,5]. Healthcare workers should have high levels of knowledge and positive attitudes toward child abuse detection. Therefore, future healthcare workers should be adequately prepared to accomplish these roles, and this preparation should start from their undergraduate studies. The limited number of studies on the awareness of child maltreatment among undergraduate healthcare students in Saudi Arabia and other parts of the globe impacts the ability to develop educational interventions to ensure a competent future healthcare workforce in determining and reporting potential child maltreatment cases.

Child abuse is a global phenomenon that affects an estimated 40 million children yearly [6]. Child abuse and neglect are defined as "any kind of physical, emotional or sexual harm to the child, causing potential or actual consequences that may affect his/her health, development or dignity" [6]. Child maltreatment is when a “parent or caregiver harms or neglects a child, resulting in possible or actual harm or threats to the child” [7]. Practices that are anchored to traditions, such as “child marriage, the caste system, gender inequality” may also have negative impacts on children and make them more vulnerable to abuse and neglect [8]. Due to cultural and societal factors that influence an individual’s perceptions and attitudes toward abuse, society members with different cultures and beliefs have varying levels of awareness regarding actions that are considered “child abuse or neglect" and the factors that lead to these actions [9].

Healthcare workers are required to account for suspected child maltreatment cases to the authorities to ensure early interventions and safeguard the child's welfare [3]. Child maltreatment is the most common problem affecting children, negatively leading to physical and mental injury [6]. Evidence indicates that child abuse is widespread, so early identification and response are vital in avoiding serious consequences among victims. Child abuse can trigger stress leading to multi-system defects. Many studies report that people who develop risky behavior or insufficient personality characteristics have a history of child abuse [10,11]. Healthcare providers are responsible for detecting and providing early care to affected children and reporting these types of cases to the relevant governments or social service agencies to take proper interventions [12].

However, previous studies have shown a lack of awareness regarding child abuse and neglect among healthcare workers who care for children. For example, Li et al. (2017) conducted a study to look into the knowledge, attitude, and behaviors to recognize, assess, and report child abuse cases among 877 healthcare providers in four regions (Hunan, Zhejiang, Shaanxi, and Guangdong) in China. The study revealed a lack of awareness, skills, and confidence regarding treating child abuse among healthcare providers [13]. The participants were also unaware of suspected cases of child abuse among their patients [13]. Another study among family physicians in Kyrgyzstan reported that most physicians surveyed had "inadequate knowledge about the identification and management of cases of child abuse” [14]. In Saudi Arabia, Alsaleem et al. (2018) evaluated the awareness level and attitude of physicians working in primary healthcare centers toward various forms of child maltreatment and assessed their actions regarding reporting and dealing with abuse cases in Abha, Saudi Arabia. They showed that most physicians showed a good level of awareness regarding child abuse and neglect [4]. However, about 64% of the physicians reported underreporting child abuse cases. Another study explored the barriers preventing pediatric nurses from reporting suspected child abuse cases at King Abdulaziz Medical City, Jeddah, Saudi Arabia [15]. The results showed that pediatric nurses would not report cases of child maltreatment due to uncertainty that the child was abused, nobody reported the suspicion, unaware of the process of reporting, and past negative experiences on the effect of reporting [15]. These studies clearly showed the need to enhance the knowledge and attitudes of healthcare workers regarding child abuse and neglect and improve their competence in determining and reporting potential cases.

In previous years, researchers worldwide have focused on studying the awareness of students from different majors (i.e., nursing, dentistry, and medicine). The results of the following studies clarify the healthcare students' insufficient knowledge and attitude about child maltreatment. For instance, Poreddi et al. (2016) conducted a study to examine the awareness and attitudes of 127 student nurses regarding child maltreatment in South India [8]. The findings indicated that nursing students lack knowledge regarding child maltreatment and negligence, but their prevention attitudes are positive. Another study in Turkey among 248 dentistry students showed that students had insufficient knowledge regarding physical and social signs of abuse [16]. In Saudi Arabia, “physical abuse and neglect” are the most prevalent types of child abuse [17]. While the awareness and reporting of suspected cases are rising, there is a lack of research that proves the awareness, knowledge, and attitude of healthcare students and healthcare workers in some parts of the country, such as in Jeddah, Saudi Arabia.

## Materials and methods

Aims

The current study evaluates the awareness of child abuse and neglect and examines the demographic factors associated with them among undergraduate healthcare students at a Saudi governmental university in Saudi Arabia.

Design

We utilized quantitative designs, specifically cross-sectional and correlational designs, to achieve our study's aims.

Sample and setting

The study’s sample comprised undergraduate healthcare students from various majors (nursing, medicine, pharmacy, dentistry, applied medical science, and medical rehabilitation science) who studied at a Saudi governmental university during the data collection. Healthcare students from all year levels were included. However, students in the preparatory, internship, and master's levels were excluded from the study. There were 2,822 healthcare students in the university during the study. With the use of the Raosoft sample size calculator (Raosoft Inc, Seattle, WA), 339 students were needed as the sample for the study at a 95% confidence level and a 5% margin of error. However, only 254 volunteered and consented to participate in our study. Out of these, 17 were excluded due to highly missing data (i.e., no answers). Hence, data from 237 students were included in the analyses.

Ethical considerations

The Research Ethics Committee at the Faculty of Nursing at King Abdulaziz University reviewed and approved the protocol of our study (Reference No.: 2B. 42). The researcher performed the investigation strictly with the ethical principles of conducting research among humans under the Declaration of Helsinki. Information about the study was provided to potential participants in the recruitment email/message. The same recruitment message contained the rights (i.e., voluntary participation and right to terminate participation), potential risks and benefits, and participation of the participants. The contact information of the principal investigator was also included in the message to address any participant's questions about the study. Electronic informed consent was solicited from the students. The students’ privacy and confidentiality were protected during the research by not collecting any information that may identify them, analyzing the data collected, and reporting the results aggregately.

Measure

An online survey using Google Forms (Google, Mountain View, CA) was used to gather the data for the study. The survey was divided into two parts, each collecting the data for the study's variables. Part 1 contained seven questions soliciting the respondents' demographics, such as age, sex, civil status, number of children, nationality, major and academic year.

Part 2 contained 23 five-point Likert scale questions to assess the awareness of the respondents on child maltreatment. This questionnaire was adapted from an earlier study by Alsaleem et al. (2018) [4]. The survey contains questions about child abuse and neglect under four categories: “Knowledge regarding child abuse and neglect” (eight questions), “Reasons for under-reporting child abuse and neglect cases in Saudi Arabia” (five questions), “Actions that are considered as child abuse” (five questions), and “Actions that are considered as child neglect” (five questions). Each question has five possible choices: “strongly agree,” “agree,” “neutral,” “disagree," and "strongly disagree.” The tool was evaluated for its “comprehensiveness, applicability, validity, and reliability” in a previous study [4]. In the study’s sample, Cronbach's alpha for the tool was 0.874, indicating acceptable reliability. The tool was initially developed in the English language. Mean scores were calculated, and high mean scores indicate higher levels of child abuse and neglect awareness.

Data collection

A recruitment message containing the survey link was distributed among the students through social media of the healthcare faculty’s official students’ accounts (i.e., WhatsApp, Twitter, and Snapchat) using a convenient sampling technique. Those interested in participating were asked to click the link, which led them to the online survey. Students who clicked "I agree" were brought to the survey, while those who clicked "I don't agree" were exited. The respondents were not given a time limit to answer the survey, but the estimated time to complete the questionnaire was between five to 10 minutes. The data were collected starting from March to May 2021. Reminders were sent/posted on the same social media accounts every two weeks until the end of the data collection period.

Statistical analysis

Data analyses were performed using IBM Statistical Package for the Social Sciences (SPSS) version 22.0 (IBM Corp., Armonk, NY). The demographic variables were treated descriptively using mean, standard deviation, frequency count, and percentage. The same analyses were performed for the data on the respondents' awareness of child maltreatment. Pearson's product correlation, independent sample t-test, and one-way analysis of variance (post hoc Tukey honest significant difference test) were conducted as appropriate to examine the association between the study variables.

## Results

A total of 237 undergraduate healthcare students were surveyed in this study (response rate = 69.9%). The age of the students ranged from 17 to 30 years (M = 21.58, SD = 1.24). The vast majority of the participants were Saudi (96.6%), females (87.8%), singles (98.3%), and had no children (99.2%). The highest proportion of the sample was from the nursing major (35.9%), while the lowest was from pharmacy and dentistry (combined percentage of 8.9%). Most students were in the 4th year of study (62.0%) (Table 1).

**Table 1:** Demographic characteristics of the respondents (n = 237)

Variable	Mean (SD)	Range
Age	21.58 (1.24)	17-30
Gender	n	%
Female	208	87.8
Male	29	12.2
Marital status		
Single	233	98.3
Married	4	1.7
With children		
No	235	99.2
Yes	2	0.8
Nationality		
Non-Saudi	8	3.4
Saudi	229	96.6
Major		
Nursing	85	35.9
Medicine	68	28.7
Applied medical science	34	14.3
Medical rehabilitation science	29	12.2
Others (pharmacy and dentistry)	21	8.9
Academic year		
2nd year	19	8.0
3rd year	68	28.7
4th year	147	62.0
5th year	2	0.8
6th year	1	0.4

Table 2 shows the findings on the students' awareness of child abuse and neglect. As reflected, the respondents’ overall average score on the scale was 82.90 (SD = 14.00) from a possible maximum score of 115. In terms of the subscales, the students rated the subscale “Actions considered as child neglect” as the highest (M = 4.18, SD = 1.08), followed by “Actions considered as child abuse” (M = 4.04, SD = 1.21), “Reasons for under-reporting child abuse and neglect cases in Saudi Arabia” (M = 3.34, SD = 0.68), and “Knowledge regarding child abuse and neglect" (M = 3.13, SD = 0.53). These results indicate a moderate awareness of child abuse and the reasons for underreporting of child maltreatment in Saudi Arabia. However, the students showed good awareness of the various forms of child maltreatment.

**Table 2:** Summary of the results of the descriptive analyses on the students’ knowledge and attitudes on child abuse and neglect (n = 237)

Variable	Mean	SD	Range	
Knowledge regarding child abuse and neglect	3.13	0.53	2.00	5.00
Reasons for under-reporting child abuse and neglect cases in Saudi Arabia	3.34	0.68	1.40	5.00
Actions considered as child abuse	4.04	1.21	1.00	5.00
Actions considered as child neglect	4.18	1.08	1.00	5.00
Overall scale mean score	3.60	0.61	1.83	5.00
Total scale score	82.90	14.00	42.00	115.00

Looking into the individual items of the scale, the percentage reporting agreement ranged from 21.5% to 81.9%. In the subscale “Knowledge regarding child abuse and neglect," five of the eight items received a very low agreement percentage. For instance, most students did not agree that they had “enough training to deal with child abuse and neglect.” Similarly, most students disagreed that "supportive services to deal with child abuse and neglect in Saudi Arabia are adequately present.” For the subscale “Reasons for under-reporting child abuse and neglect cases in Saudi Arabia,” most of the students did not agree on the following reasons: “it is not legally mandated to report child abuse” and “reporting might not be good for the sake of the child.” However, most students agreed to all the items on the actions considered as child abuse and child neglect. The complete findings on this area are presented in Table 3.

**Table 3:** Agreement and disagreement of the respondents on the scale’s item (n = 237)

Items	Agree/strongly agree, n (%)	Neutral, n (%)	Disagree/strongly disagree, n (%)
Knowledge regarding child abuse and neglect			
1. I have enough training to deal with child abuse and neglect.	51 (21.5)	75 (31.6)	111 (46.8)
2. I think we need to redefine child abuse and neglect in Saudi Arabia according to our culture and religion.	175 (73.8)	34 (14.3)	28 (11.8)
3. I think that supportive services to deal with child abuse and neglect in Saudi Arabia are adequately present.	82 (34.6)	70 (29.5)	85 (35.9)
4. I prefer resolving a case of child abuse myself rather than reporting it to the police.	54 (22.8)	69 (29.1)	114 (48.1)
5. I am aware of the reporting procedure for child abuse in my community in Saudi Arabia.	71 (30.0)	47 (19.8)	119 (50.2)
6. I am willing to report all suspected child abuse cases.	141 (59.5)	55 (23.2)	41 (17.3)
7. I prefer to limit my reporting about child abuse to life-threatening injuries.	51 (21.5)	59 (24.9)	127 (53.6)
8. I think child abuse is under-reported in Saudi Arabia.	165 (69.6)	46 (19.4)	26 (11.0)
Reasons for under-reporting child abuse and neglect cases in Saudi Arabia			
9. It is not legally mandated to report child abuse.	56 (23.6)	66 (27.8)	115 (48.5)
10. Reporting might not be good for the sake of the child.	78 (32.9)	63 (26.6)	96 (40.5)
11. Reporting procedures are unclear.	124 (52.3)	70 (29.5)	43 (18.1)
12. Reporting child abuse to authorities is not yet acceptable in our community.	139 (58.6)	51 (21.5)	47 (19.8)
13. Fear of parent response.	185 (78.1)	37 (15.6)	15 (6.3)
Actions considered as child abuse			
14. Burning the child for misbehaviors.	180 (75.9)	23 (9.7)	34 (14.3)
15. Locking the child alone at home.	170 (71.7)	21 (8.9)	46 (19.4)
16. Severe beating that leaves marks on the child's body.	189 (79.7)	10 (4.2)	38 (16.0)
17. Parents throwing different objects at the child when angry.	182 (76.8)	25 (10.5)	30 (12.7)
18. Parents who smoke in the presence of the child.	159 (67.1)	43 (18.1)	35 (14.8)
Actions considered as child neglect			
19. Parents refused to send the child to school.	189 (79.7)	21 (8.9)	27 (11.4)
20. Parents refused medical treatment or surgical intervention necessary for their child.	187 (78.9)	23 (9.7)	27 (11.4)
21. A child with severe dental problems, which are not treated.	180 (75.9)	27 (11.4)	30 (12.7)
22. Parents pay no attention to the child's cleanliness.	194 (81.9)	19 (8.0)	24 (10.1)
23. The child fails to thrive due to social deprivation.	183 (77.2)	36 (15.2)	18 (7.6)

Statistical tests were conducted to examine the students' demographics associated with their awareness level (all subscales). The analyses showed that gender (t = -3.88, p < 0.001) and year level (F = 5.98, p = 0.003) were associated with the students' "knowledge regarding child abuse and neglect." Specifically, male students (M = 3.48, SD = 0.51) have statistically higher knowledge in this area than female students (M = 3.09, SD = 0.51). Also, 2nd-year students (M = 3.53, SD = 0.59) had statistically higher knowledge in this area than students in the 3rd (M = 3.12, SD = 0.60; p = 0.007) and 4th year (M = 3.09, SD = 0.47; p = 0.002). No statistically significant associations were observed between the other demographic variables and the students’ awareness subscales.

Finally, Table 4 reflects that the four subscales of the study instrument had low to high positive correlations, indicating that these variables are interrelated.

**Table 4:** Correlation between the study’s variables (n = 237) * Significant at 0.05 level. ** Significant at 0.01 level. *** Significant at 0.001 level.

Variables	Knowledge regarding child abuse and neglect	Reasons for under-reporting child abuse and neglect cases in Saudi Arabia	Actions considered as child abuse
Reasons for under-reporting child abuse and neglect cases in Saudi Arabia	r = 0.31 (p < 0.001***)		
Actions considered as child abuse	r = 0.31 (p = 0.002**)	r = 0.29 (p < 0.001***)	
Actions considered as child neglect	r = 0.15 (p = 0.020*)	r = 0.24 (p < 0.001***)	r = 0.82 (p < 0.001***)

## Discussion

Children are vulnerable to abuse and the possibilities for such abuse are limitless. Abuse may occur in every country and place where children should be the most protected in their surrounding environment. Children's rights violation is considered one of the most critical concerns around the world. Based on the United Nations International Children's Emergency Fund (UNICEF, 2021) child maltreatment report, child abuse’s general nature and impact have become increasingly acknowledged over the last decade [18]. Thus, this study was conducted to assess child maltreatment awareness among healthcare students in Jeddah, Saudi Arabia. Our study provided valuable findings on preparing future healthcare workers to provide care to pediatric patients who are victims of maltreatment.

The findings of the current study showed a decent awareness of child maltreatment, as reflected by the overall average score of 89.90 (SD = 14.00) from the possible maximum score of 115. This score represents 72.1%, more than 50% of the total score, implying that the respondents are aware of the issues surrounding child abuse and neglect. Previous studies reported similar findings. For instance, a previous study conducted among medical students in Dammam, Saudi Arabia, reported that most of the students were aware of child abuse, that these issues existed in their locality, and perceived the critical roles of doctors in interventions related to the issue [19]. However, previous studies showed inadequate knowledge and attitudes toward child abuse among Indian student nurses and dentistry students in Turkey [8,16]. Considering these students are the future healthcare workforce in the country, they should be trained to act competently when they encounter patients who are victims of abuse. Previous studies in the country highlight the need to enhance the education of healthcare students on this matter to ensure a future competent healthcare workforce [19,20].

The scores of our sample were high on the subscales “actions considered as child neglect” and “actions considered as child abuse." These findings indicate that the surveyed healthcare students were aware of the various actions that constitute abuse and negligence for pediatric patients. For instance, the students were fully aware that actions such as burning the child, locking the child, severely beating the child, throwing different objects, and smoking in front of the child constitute child abuse. The students were also fully aware that parents' “refusal to send the child to school, refusal to seek medical treatment for the child, and paying no attention to the child,” as well as children having dental problems and failing to thrive, signify actions of child neglect.

However, the students in our study reported lower scores in the dimension of “reasons for under-reporting child abuse and neglect cases in Saudi Arabia.” This indicates that the students were not fully aware of the different reasons for the under-reporting of child maltreatment cases in the country. Tiyyagura et al. (2017) reported "fear of being wrong; fear of caregiver reactions; and working in a fast-paced setting" as barriers to under-reporting of cases of child maltreatment [21]. A narrative review performed by Azizi and Shahhosseini (2017) found that a combination of individual factors (i.e., knowledge, attitudes and beliefs, lack of experience experiences, and uncertain diagnoses), interpersonal factors (i.e., fear and breach of confidentiality), organizational factors (i.e., communication problems and poor legal reporting processes), and situational factors (i.e., characteristics and evidence) is a reason for not reporting cases among healthcare workers [22]. The low awareness of healthcare students for these reasons may add to their challenges when practicing their profession. Lack of awareness of these reasons may also cause these future healthcare workers to under-report suspected cases of child abuse, which can result in worse scenarios and long-term effects, such as continuance of the child maltreatment, and "increased risk for several problematic developmental, health, and mental health outcomes” [23]. Therefore, these findings call for the improvement of the student's awareness in this area. Healthcare students should be educated about the different reasons for under-reporting child abuse cases, not only in the country but worldwide.

Moreover, the results showed lower scores in the dimension “knowledge regarding child abuse and neglect.” The finding showed that the students thought they did not have enough training to intervene in cases of child abuse. They also thought there were inadequate services in place in the country that support dealing with cases of child maltreatment. There was also a low percentage of healthcare students who were aware of the “child abuse reporting system” in the country and a low percentage of students who thought of resolving the case themselves rather than reporting it to authorities. Alarmingly, more students preferred to report only child abuse cases with life-threatening injuries. Our respondents' poor awareness of these areas is also reflected in previous studies in the country. For instance, Alsaleem et al. reported that even practicing physicians in the country perceived that they had limited training on this matter [4]. They also would like to redefine “child abuse and neglect” in the country based on their culture and religion. The study also reported that the physicians thought of a lack of services in the country to tackle child maltreatment. Our findings and the findings of Alsaleem et al. underscore the necessity to strengthen education on child maltreatment among healthcare students in the country [4]. The poor awareness of healthcare students and physicians in this matter calls for a review of the course contents that tackle these topics. Healthcare students should be adequately taught about child maltreatment, their roles, and the issues surrounding it in their undergraduate education to ensure that they possess the necessary knowledge, skills, and attitudes to intervene with child abuse in their future practice.

The tests of associations of the study variables signified that male students had higher scores in the dimension “knowledge regarding child abuse and neglect" than female students. Previous studies showed no gender differences in the knowledge of child maltreatment among healthcare workers [4]. The study of Al-Qahtani et al. (2022) showed contrasting results where, according to this study, a more significant percentage of female medical students reported child maltreatment is common in Saudi Arabia [19]. Also, in the same study, significantly more female students believed physicians have critical roles in dealing with child maltreatment and preferred to receive more education about this topic than male students. The inconsistent findings reported in our findings and the previous literature underscore the need for further investigation. Our findings may have been influenced by the significantly lower proportion of male students than female students, hence, the difference in the awareness level. Therefore, future studies can examine the gender difference in healthcare students' “awareness, knowledge, and attitudes toward child abuse and neglect.” Moreover, our findings also showed significantly higher scores in this dimension among 2nd-year students than 3rd and 4th-year students. It is difficult to identify the reason behind this finding, considering the differences in the curriculum and courses between the different majors. For instance, the nursing program tackles the topic of child abuse in courses such as pediatric nursing and community health nursing. These courses are usually in the latter years of the program. Other programs have this topic integrated into courses in different year levels. Al-Qahtani et al. (2022) reported different levels of awareness of child maltreatment among students. However, their investigation did not explain the reasons for those differences [19].

Limitations

While the research provided additional information on future healthcare workers' awareness of child maltreatment, the study has some recognized limitations that need to be considered. First, the study's sample size is small, and the sampling technique used was a convenience sample due to time limitations. Because of this, the study's findings are limited to the population and setting and should not be generalized to the healthcare students in the country. Second, the factors associated with the student's awareness of the topics were limited to a few demographic profiles. Future studies should consider other factors based on the literature. Third, the study did not explore the reasons behind the differences in awareness between gender and year of study. Future studies could be focused on exploring the reasons through qualitative inquiry. Nonetheless, the study’s results contributed to the literature on child abuse and neglect awareness among healthcare students in the country and the Arab region.

Implications

The findings provided evidence of the awareness of healthcare students regarding “child abuse and neglect.” The findings support the enhancement of the curriculum of the healthcare programs in the country to ensure that topics on “child abuse and neglect” are adequately covered. Policymakers in the healthcare education sector can use the findings in this study to identify the areas that need to be included in the healthcare education curriculum. For instance, the findings identified that the students needed to be fully aware of the reasons for the under-reporting of cases in the country. Therefore, topics on the barriers and facilitators of reporting cases among healthcare workers should be included in the curriculum. Also, in dealing with these topics, the local context (i.e., in Saudi Arabia), such as the culture, religion, society, policies, beliefs, and protocols, should be considered. Furthermore, faculty members can use the findings in designing their courses (theoretical or clinical) to ensure that topics on child abuse and neglect are integrated.

## Conclusions

The study focused on assessing healthcare students' awareness of child maltreatment in a Saudi Arabian university. Findings revealed good awareness of “child abuse and neglect” among healthcare students. Specifically, the students in this study showed good awareness of the different actions that can be considered abusive and negligent to a child. However, the healthcare students in this study reported lower awareness of the different reasons for the country's under-reporting of child maltreatment cases and insufficient knowledge of the concepts of “child abuse and neglect.” The findings also suggest differences in “child abuse and neglect” knowledge between genders and academic levels; however, we recommend future studies to verify such differences. The findings call for the review and improvement of the courses of the healthcare program to ensure that topics on “child abuse and neglect” are adequately covered.
